# Cervical Spinal Cord Atrophy can be Accurately Quantified Using Head Images

**DOI:** 10.1177/20552173211070760

**Published:** 2022-01-07

**Authors:** Kamyar Taheri, Irene M Vavasour, Shawna Abel, Lisa Eunyoung Lee, Poljanka Johnson, Stephen Ristow, Roger Tam, Cornelia Laule, Nathalie Chantal Ackermans, Alice Schabas, Helen Cross, Jillian Katrina Chan, Ana-Luiza Sayao, Virender Bhan, Virginia Devonshire, Robert Carruthers, David KB Li, Anthony L Traboulsee, Shannon H Kolind, Adam Vladimir Dvorak

**Affiliations:** Department of Medicine, 8166 University of British Columbia, Vancouver, BC, Canada; Department of Radiology, 8166 University of British Columbia, Vancouver, BC, Canada; Department of Medicine, 8166 University of British Columbia, Vancouver, BC, Canada; Department of Radiology, 8166 University of British Columbia, Vancouver, BC, Canada; Department of Radiology, 8166 University of British Columbia, Vancouver, BC, Canada; Department of Pathology & Laboratory Medicine, 8166 University of British Columbia, Vancouver, BC, Canada; Department of Physics & Astronomy, 8166 University of British Columbia, Vancouver, BC, Canada; International Collaboration on Repair Discoveries (ICORD), Vancouver, BC, Canada; Department of Medicine, 8166 University of British Columbia, Vancouver, BC, Canada; Department of Medicine, 8166 University of British Columbia, Vancouver, BC, Canada; Department of Radiology, 8166 University of British Columbia, Vancouver, BC, Canada; Department of Medicine, 8166 University of British Columbia, Vancouver, BC, Canada; Department of Medicine, 8166 University of British Columbia, Vancouver, BC, Canada; Department of Radiology, 8166 University of British Columbia, Vancouver, BC, Canada; Department of Physics & Astronomy, 8166 University of British Columbia, Vancouver, BC, Canada; International Collaboration on Repair Discoveries (ICORD), Vancouver, BC, Canada; Department of Physics & Astronomy, 8166 University of British Columbia, Vancouver, BC, Canada; International Collaboration on Repair Discoveries (ICORD), Vancouver, BC, Canada

**Keywords:** Spinal cord, magnetic resonance imaging, multiple sclerosis, atrophy, neuroimaging, cervical cord

## Abstract

**Background:**

Spinal cord atrophy provides a clinically relevant metric for monitoring MS. However, the spinal cord is imaged far less frequently than brain due to artefacts and acquisition time, whereas MRI of the brain is routinely performed.

**Objective:**

To validate spinal cord cross-sectional area measurements from routine 3DT1 whole-brain MRI versus those from dedicated cord MRI in healthy controls and people with MS.

**Methods:**

We calculated cross-sectional area at C1 and C2/3 using T2*-weighted spinal cord images and 3DT1 brain images, for 28 healthy controls and 73 people with MS. Correlations for both groups were assessed between: (1) C1 and C2/3 using cord images; (2) C1 from brain and C1 from cord; and (3) C1 from brain and C2/3 from cord.

**Results and Conclusion:**

C1 and C2/3 from cord were strongly correlated in controls (*r* = 0.94, *p*<0.0001) and MS (*r* = 0.85, *p*<0.0001). There was strong agreement between C1 from brain and C2/3 from cord in controls (*r* = 0.84, *p*<0.0001) and MS (*r* = 0.81, *p*<0.0001). This supports the use of C1 cross-sectional area calculated from brain imaging as a surrogate for the traditional C2/3 cross-sectional area measure for spinal cord atrophy.

## Introduction

Multiple Sclerosis (MS) is an inflammatory, demyelinating disease that causes neurodegeneration in the central nervous system. Cord atrophy measurements from magnetic resonance imaging (MRI)^1^ provide a valuable and clinically relevant metric for monitoring MS.^2^ The C2/3 segment of the cervical cord is often used to assess atrophy in MS due to its high concentration of lesions, increased rate of atrophy, and relative technical ease compared to assessment of the whole cord.^3–5^

Spinal cord is imaged far less frequently than brain due to artefacts and acquisition time.^1,6^ 3-dimensional, T1-weighted (3DT1) brain images, which are recommended for volumetric assessment of MS brain,^7^ often capture a few upper cervical spinal cord levels, depending on the field-of-view (FOV). If C1 cross-sectional area (CSA) accurately reflects C2/3 CSA, which has been established as clinically relevant, and CSA measurements derived from brain images agree with spinal cord image values, then a clinically relevant measure of spinal cord atrophy could be obtained using only a standard 3DT1 brain image. Previously, Liu et al. showed strong agreement between upper cervical cord area values calculated from 3D T1-weighted images of the head and cord.^8^ In 2018, Papinutto et al. demonstrated that reproducible upper cervical cord area measurements can be derived from head images, so long as corrections for gradient non-linearities are performed.^9^

The overall aim of this study was to determine if measurements of C1 CSA, derived from 3DT1 brain images, can provide a surrogate measurement for C2/3 CSA measurements obtained from dedicated spinal cord imaging. All groupwise analyses were performed for both healthy controls and people with MS. We first investigated the relationship between C1 CSA calculated from cord images versus C2/3 CSA calculated from cord images. We then assessed the agreement between C1 CSA calculated from brain images and C1 CSA calculated from cord images. Finally, we directly assessed the relationship between C1 CSA calculated from brain images (our proposed cord atrophy surrogate measure) and C2/3 CSA calculated from spinal cord images. This work builds upon previous literature by 1) directly comparing values from standard 3DT1 brain imaging to those from multi-gradient echo T2*-weighted cord imaging, recommended by the International Spinal Cord Society (ISCoS)^10^ and 2) maximizing applicability by using only default vendor-implemented gradient non-linearity corrections and processing with Spinal Cord Toolbox (SCT),^11,12^ the standard for cord atrophy analysis.

## Materials and methods

### Participants

The study was approved by the University of British Columbia Clinical Research Ethics Board. All participants provided written informed consent prior to participation. We collected data from 28 healthy controls (11 M/17 F, mean age 47 years, age range 22–65 years) and 73 people with clinically definite MS, fulfilling the 2017 revised McDonald criteria for diagnosis,^13^ (25 M/48 F, mean age 50 years, age range 26–65 years, 38 relapsing-remitting MS (RRMS); 12 primary progressive MS (PPMS); 23 secondary progressive MS (SPMS), median Expanded Disability Status Scale (EDSS)^14^ 3.5, EDSS range 1.0–8.5, median disease duration 12.0 years, disease duration range 0.3–48 years). Control and MS groups did not differ significantly in age or sex (*p* > 0.10). De-identified data will be shared following a reasonable request by a qualified investigator.

### Imaging

Data was collected at the UBC MRI Research Centre on a 3.0 Tesla MRI scanner (Achieva, Philips Healthcare, Best, The Netherlands). For brain imaging, a 3D T1-weighted magnetization-prepared rapid gradient-echo sequence was acquired (resolution 1 × 1 × 1 mm, FOV = 256 × 256 × 165 mm, repetition time (TR) = 8.1 ms, echo time (TE) = 2.5 ms, inversion time (TI) = 1052 ms, shot interval = 3000 ms, acquisition time = 6 min 26 s) using an 8-channel head coil. For spinal cord imaging, a T2*-weighted multi-echo rapid gradient echo scan was collected (resolution 0.8 × 0.8 × 2.5 mm, FOV = 150 × 150 × 44 mm, TR = 815 ms, TE = 6.5 ms, ΔTE = 8.2 ms, 5 echoes (cumulated on the scanner), acquisition time = 5 min 7 s) using a 6-channel spine coil with the FOV centred at the level of the C2/3 disc. Brain and spinal cord data was collected during the same scan session. All images were reconstructed including the default vendor-implemented gradient non-linearity corrections.

### Analysis

SCT^11,12^ was used to (1) automatically segment the spinal cord^15^, (2) label cord vertebral levels using the manually identified C2/3 disc and image intensity of the vertebral columns and intervertebral discs, and (3) extract the mean cord CSA using the SCT segmentation across all image slices contained in the C1 level and in the C2/3 levels. CSA derived from brain images is abbreviated as CSA_brain_ and CSA derived from spinal cord images is abbreviated as CSA_cord_. Manual quality control was performed to ensure accuracy of the cord segmentation and labelling of the cord vertebral levels.

All statistical analyses were performed for both control and MS groups. We compared: **(1) C1 CSA_cord_ versus C2/3 CSA_cord_**, to check for underlying correspondence between CSA at these different anatomical locations, **(2) C1 CSA_brain_ versus C1 CSA_cord_**, to assess the agreement between CSA calculated for the same location from standard brain 3DT1 images and from ISCoS recommended T2*-weighted cord images, and **(3) C1 CSA_brain_ versus C2/3 CSA_cord_**, to provide a direct validation of the ability to use C1 CSA_brain_ as a surrogate for C2/3 CSA_cord_. For all comparisons, Spearman correlation coefficients (r) and 95% confidence intervals were calculated for relationships between CSA_cord_ or CSA_brain_ at different levels. Linear regression was used to model the relationships between CSA measures. Finally, Bland-Altman analysis was used to assess their agreement accounting for bias. For C1 CSA_brain_ versus C1 CSA_cord_, the only comparison between paired values of the same measure, Wilcoxon paired t-tests were used to assess the agreement between CSA_brain_ and CSA_cord_, presented as means with 95% confidence intervals. We chose to use non-parametric analyses due to our sample size, especially for the healthy control group with fewer than 30 subjects, because 30 is the minimum number suggested for analyses assuming a parametric distribution. All statistical analysis was done using GraphPad Prism 7.

## Results

### C1 CSA_cord_ versus C2/3 CSA_cord_

#### Controls

**Figure 1.**
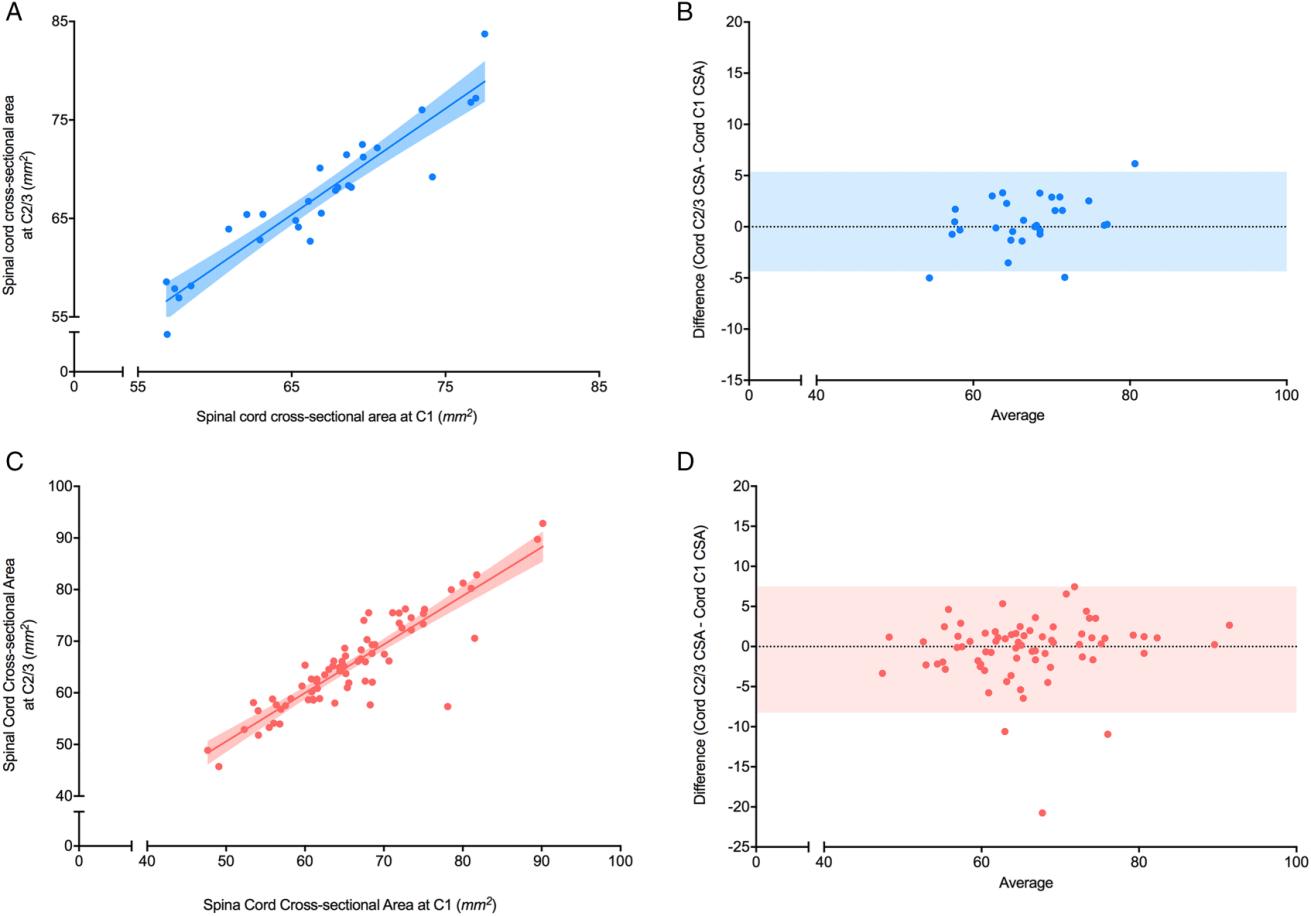
Correlation between C1 CSA with C2/3 CSA from cord imaging in healthy controls and people with multiple sclerosis (MS). (A) Correlation between spinal cord cross-sectional area (CSA) (mm^2^) from healthy controls measured using spinal cord imaging at C1 and at C2/3 (*n* = 28). The r value was 0.94 with *p*<0.0001. A linear regression model was used to fit the data with r^2^ value of 0.88, slope of 1.1, and y-intercept of −4.6 mm^2^. The shaded areas represent the 95% confidence intervals. (B) Bland-Altman plot between spinal cord CSA (mm^2^) from healthy controls measured using cord imaging at C1 and at C2/3. A bias of 0.50 mm^2^ and 95% limits of agreement (shaded region) from −4.4 to 5.4 mm^2^ were found. (C) Correlation between spinal cord CSA (mm^2^) measured from people with MS measured at C1 and at C2/3 using cord imaging (*n* = 73). The r value was 0.85 with *p*<0.0001. A linear regression model was used to fit the data with *r*^2^ = 0.80, slope = 0.94, and y-intercept = 3.6 mm^2^. The shaded area represents the 95% confidence interval. (D) Bland-Altman plot for spinal cord CSA (mm^2^) measured from people with MS at C1 and at C2/3 using cord images. A bias of −0.38 mm^2^ and a 95% limit (shaded region) of agreement from −8.2 to 7.5 mm^2^ was found.

C1 CSA_cord_ was positively correlated with C2/C3 CSA_cord_ in the control group (*r* = 0.94 (0.89–0.97), *p*<0.0001, Figure 1A) with a linear regression model slope of 1.1 and y-intercept of −4.6 mm^2^ (*r*^2^ = 0.88). Bland-Altman analysis showed a bias of 0.50 mm^2^, a 95% limit of agreement of −4.4 to 5.4 mm^2^, and no significant non-zero slope (*p* = 0.052; Figure 1B).

#### Multiple sclerosis

C1 CSA_cord_ was positively correlated with C2/C3 CSA_cord_ in people with MS (*r* = 0.85 (0.77–0.91), *p*<0.0001, Figure 1C) with a linear regression model slope of 0.94 and y-intercept of 3.6 mm^2^ (r^2^ of 0.80). Bland-Altman analysis showed a bias of −0.38 mm^2^, a 95% limit of agreement of −8.2 to 7.5 mm^2^ and no significant non-zero slope (*p* = 0.37; Figure 1D).

### C1 CSA_brain_ versus C1 CSA_cord_

#### Controls

**Figure 2.**
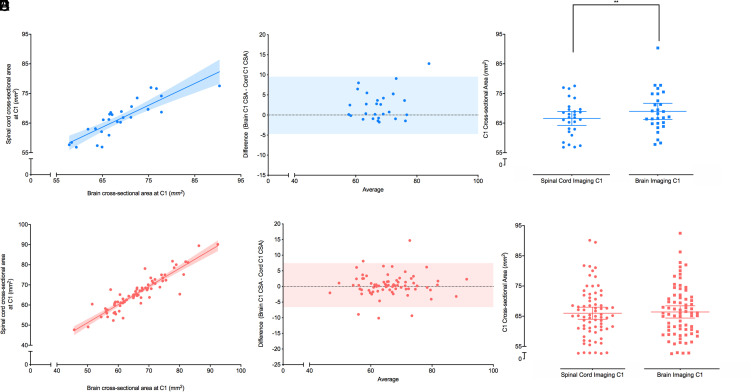
Correlation between C1 CSA from brain imaging with C1 CSA from cord imaging in healthy controls and people with multiple sclerosis (MS). (A) Correlation between spinal cord cross-sectional area (CSA) (mm^2^) from healthy controls measured at C1 using T1-weighted brain imaging and T2*-weighted cord imaging. The r value was 0.89 with *p*<0.0001. A linear regression model was used to fit the data with r^2^ value of 0.73, slope of 0.74, and y-intercept of 16 mm^2^. The shaded area represents the 95% confidence interval (*n* = 28). (B) Bland-Altman plot between spinal cord CSA (mm^2^) from healthy controls measured at C1 using brain imaging and cord imaging. A bias of 2.4 mm^2^ and 95% limits of agreement (shaded region) from −4.7 to 9.6 mm^2^ were found. (C) Grouped analysis of spinal cord CSA (mm^2^) from healthy controls measured at C1 using cord (circles) and brain (squares) imaging. The spinal cord CSA had a mean of 66 (64–69) mm^2^ and the brain C1 CSA had a mean of 69 (66–72) mm^2^. Mean and 95% confidence intervals of each group are shown. A non-parametric Wilcoxon paired t-test showed a significant mean difference of 2.4 (1.0–3.8) mm^2^ with *p* = 0.0041. (D) Correlation between C1 CSA (mm^2^) from people with MS measured using T1-weighted brain imaging and T2*-weighted cord imaging. The r value was 0.91 with *p*<0.0001. A linear regression model was used to fit the data with r^2^ value of 0.83, slope of 0.90, and y-intercept of 6.2 mm^2^. The shaded area represents the 95% confidence interval (*n* = 73). (E) Bland-Altman plot between C1 CSA (mm^2^) from people with MS measured using brain imaging and cord imaging. A bias of 0.43 mm^2^ and 95% limits of agreement (shaded region) from −6.6 to 7.5 mm^2^ were found (*n* = 73). (F) Grouped analysis of spinal cord CSA (mm^2^) from people with MS measured at C1 using cord (circles) and brain (squares) imaging. The spinal cord CSA had a mean of 66 (64–68) mm^2^ and the brain C1 CSA had a mean of 66 (64–68) mm^2^. Mean and 95% confidence intervals of each group are shown. A non-parametric Wilcoxon paired t-test for the mean difference of 0.4 mm^2^ was not significant with *p* > 0.2.

The relationship between C1 CSA_cord_ and C1 CSA_brain_ showed a positive correlation (*r* = 0.89 (0.78–0.95), *p*<0.0001, Figure 2A) with a linear regression model slope of 0.74 and a y-intercept 16 mm^2^ (*r*^2^ = 0.73). Bland-Altman analysis showed a bias of 2.4 mm^2^, a 95% limit of agreement of −4.7 to 9.6 mm^2^ and no significant non-zero slope (*p* = 0.16, Figure 2B). A non-parametric t-test showed a small but significant difference of 2.4 mm^2^ (1.0–3.8 mm^2^, *p* = 0.0041) between C1 CSA_cord_ (mean = 66 mm^2^, 64–69 mm^2^ 95% CI) and C1 CSA_brain_ (mean = 69 mm^2^, 66–72 mm^2^ 95% CI) in the control group (Figure 2C).

#### Multiple sclerosis

The relationship between C1 CSA_cord_ to C1 CSA_brain_ for MS also showed a positive correlation (*r* = 0.91 (0.86–0.94), *p*<0.0001, Figure 2D) with a linear regression model slope of 0.90 and a y-intercept of 6.2 mm^2^ (*r*^2^ = 0.83). Bland-Altman analysis showed a bias of 0.43 mm^2^, a 95% limit of agreement of −6.6 to 7.5 mm^2^, and no significant non-zero slope (*p* = 0.78, Figure 2E). A non-parametric t-test for the mean difference of 0.4 mm^2^ was not significant (*p* > 0.2) between C1 CSA_cord_ (mean = 66 mm^2^, 64–68 mm^2^ 95% CI) and C1 CSA_brain_ (mean = 66 mm^2^, 64–68 mm^2^ 95% CI) in people with MS (Figure 2F).

### C1 CSA_brain_ versus C2/3 CSA_cord_

#### Controls

**Figure 3.**
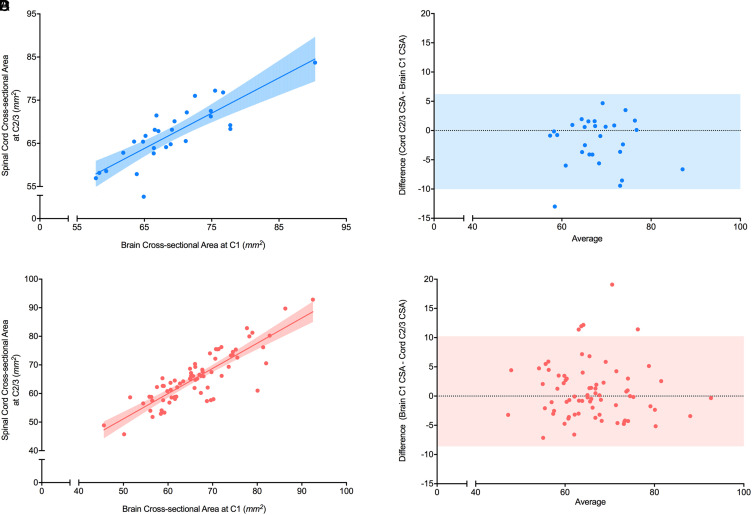
Correlation between C1 CSA from brain imaging and C2/3 CSA from cord imaging in healthy controls and people with multiple sclerosis (MS). A) Correlation between spinal cord cross-sectional areas (CSA) (mm^2^) measured from healthy controls at C1 using brain imaging and at C2/3 using cord imaging. The r value was 0.84 with *p*<0.0001. A linear regression model was used to fit the data with *r*^2^ value of 0.68, slope of 0.82, and y-intercept of 11 mm^2^. The shaded area represents the 95% confidence interval (*n* = 28). (B) Bland-Altman plot for spinal cord CSA (mm^2^) measured from healthy controls at C1 using brain images and at C2/3 using cord images. A bias of −1.9 mm^2^ and 95% limits of agreement (shaded region) from −10 to 6.2 mm^2^ were found. (C) Correlation between CSA (mm^2^) measured from people with MS at C1 using brain imaging and at C2/3 using cord imaging. The r value was 0.81 with *p*<0.0001. A linear regression model was used to fit the data with r^2^ value of 0.73, slope of 0.88, and y-intercept of 7.1 mm^2^. The shaded area represents the 95% confidence interval (*n* = 73). (D) Bland-Altman plot for spinal cord CSA (mm^2^) measured from people with MS at C1 using brain imaging and at C2/3 using cord imaging. A bias of −0.81 mm^2^ and 95% limits of agreement (shaded region) from −10 to 8.6 mm^2^ were found (*n* = 73).

C1 CSA_brain_ was positively correlated with C2/3 CSA_cord_ in healthy individuals (*r* = 0.84 (0.68–0.92), *p*<0.0001, Figure 3A) with a linear regression model slope of 0.82 and y-intercept 11 mm^2^ (*r*^2^ = 0.68). Bland-Altman analysis showed a bias of −1.9 mm^2^, a 95% limit of agreement of −10 to 6.2 mm^2^ and no significant non-zero slope (*p* = 0.095, Figure 3B).

#### Multiple sclerosis

C1 CSA_brain_ was also positively correlated with C2/3 CSA_cord_ in people with MS (*r* = 0.81 (0.70–0.88), *p*<0.0001, Figure 3C) with a linear regression model slope of 0.88 and y-intercept 7.1 mm^2^ (*r*^2^ = 0.73). Bland-Altman analysis showed a bias of −0.81 mm^2^, a 95% limit of agreement of −10 to 8.6 mm^2^ and no significant non-zero slope (*p* = 0.59, Figure 3D).

## Discussion

Strong relationships were found between all CSA measurements in both healthy controls and people with MS, obtained from spinal cord and brain images. Since C2/3 CSA is more commonly utilized in cord analysis and has been associated with disability as well as disease progression,^16–21^ we first assessed whether high resolution spinal cord image CSA measurement at C1 (which can typically be assessed from brain images) yielded values consistent with those from C2/3. The strong linear correlation and even distribution of data points around the bias in the Bland-Altman analysis with non-significant slope support the use of C1 measurements as a suitable proxy for more commonly used C2/3 CSA in cord atrophy analysis. Although C2/3 is included in some brain MRI, it lies close to the edge of the FOV and can be affected by gradient non-linearities.^9^ Furthermore, C2/3 is not as commonly or consistently covered in typical patient brain exams which could lead to biased results and reduced statistical significance.

Having established that C1 CSA can be used in place of C2/3 CSA, the consistency of C1 CSA measurements between dedicated spinal cord imaging and brain images was assessed. Once again, strong correlations and even distribution of data points around the bias with non-significant slope (*p* = 0.16) were demonstrated. The strong agreement between CSA measurements implies that dedicated cord imaging may not be necessary for quantitative measurements of spinal cord atrophy, and that 3DT1 brain scans can provide the same information.

A small bias was observed when comparing the two values in healthy controls. A potential cause of this bias could be gradient non-linearities, however this is unlikely as the FOV of most brain images extends to C4 in the inferior caudal direction. Furthermore, the brain images have a sagittal readout with frequency encoding in the superior-inferior/foot-head direction, meaning that the superior-inferior FOV edges will be less affected by gradient non-linearities than in the right-left or anterior-posterior directions. A more likely reason could be the difference in contrast between T1- and T2*-weighted images causing different cord segmentation boundaries. The bias was not found in the larger group of people with MS and is inconsequential so long as measurements are taken from a consistent image contrast and that measurements from different image contrasts are not mixed.

Image resolution will also influence cord CSA measurement. Smaller voxels have improved accuracy due to less bias imposed by the method of thresholding the cord segmentation in the presence of partial volume effects, in voxels containing signal from both cord and cerebrospinal fluid. Reduced partial volume effects will be especially evident for smaller voxels in the superior-inferior direction, such as those acquired with a 3D isotropic acquisition, particularly in cases where the cord cannot be aligned perpendicularly to the superior-inferior imaging plane throughout the FOV.^22^ Higher superior-inferior resolution will also improve the precision of cord CSA measurements, since CSA values will be averaged from more slices within each vertebral level and will therefore be less subject to the effects of outliers or the natural variation in size of the cervical cord along its length. Of course, the benefits of higher resolution acquisition come at the cost of longer acquisition times, making the images more prone to motion artefacts. Although determining the optimal sequence and resolution for CSA measurement is not trivial, high resolution isotropic 3D acquisitions are more easily accommodated by T1-weighted brain imaging sequences than by T2*-weighted cord imaging sequences (due to the much shorter TR and less difficult imaging environment), an additional benefit to calculating CSA from 3DT1 brain images.

Finally, we directly compared C1 CSA taken from brain images to C2/3 CSA measured from dedicated spinal cord imaging and found a strong relationship in both healthy controls (*r* = 0.84) and MS (*r* = 0.81). Taken all together, this study demonstrated that C1 CSA derived from brain images largely reflects C2/3 CSA measurements derived from cord images. This finding suggests that, when combined with the latest spinal cord image analysis tools, brain imaging can provide surrogate measures for cord atrophy. Furthermore, we observed minimal bias and a linear response reflecting changes in cord C2/3 CSA. This agrees with previous studies done by Liu et al. who showed that measurement at 2.5 cm below the pons in brain images is an appropriate surrogate for upper cord atrophy using semi- automated NeuroQLab software (*n* = 97), and Papinutto et al. who showed excellent C2-C3 area reliability when derived from brain scans using the semi-automated “cord finder option” in the JIM analysis software, which required multiple marker inputs (*n* = 3).^8,9^ The current guidelines set by the ISCoS recommend the use of multi-gradient echo T2*-weighted scans centred at C2/3 for imaging and the use of JIM software or SCT for analysis of spinal cord atrophy.^10^ Previous work in this area did not include a direct comparison to T2*-weighted cord imaging focused on validated C2/3 for measurement of CSA, or did not have a large sample size. Furthermore, our analysis used SCT, which is openly available and quickly becoming the standard for spinal cord image analysis. Our results are the first direct validation for the use of C1 CSA acquired from standard 3DT1 brain imaging as a surrogate for the C2/3 multi-gradient echo T2*-weighted cord-acquired CSA, as recommended by ISCoS, in the quantification spinal cord atrophy in people with MS.

### Limitations

The specificity of CSA to pathological atrophy is one limitation of this metric. Processes such as inflammation^5^ may confound results in people with MS. This may explain the slightly lower correlation coefficients between C1 and C2/3 measurements seen in people with MS. Furthermore, usage of C1 as surrogate for C2/3 will likely not suffice in studies investigating the structural-functional relationships specific to C2/3.

The images for this study were acquired from a single site which limits the generalizability of the study. However, use of only a single site avoids the confound of variability between scanners and scan parameters during initial assessment of the surrogate cord atrophy measure.

Structural variability is increased in the cervical region for people with MS which makes comparison between individuals difficult.^18^ Normalization to characteristics such as spinal cord length or participant height can be used to help remedy this issue, although some studies have shown that un-normalized values can provide increased sensitivity.^23^ Alternatively, some studies advocate for newer slicewise normalization techniques.^24^ Although our study circumvented the problem of inter-subject structural variability by comparing between acquisitions for the same individual, future studies investigating this issue in the context of CSA measurements derived from brain images would be valuable. Finally, as this study was cross-sectional, we cannot comment on how sensitive brain imaging-derived C1 CSA will be to changes in spinal cord imaging-derived C2/C3 CSA over time. Longitudinal acquisition is needed for comparison of changes in CSA over time between the different cord levels and image contrasts.

### Significance and implications

The spinal cord plays an important role in MS disability and progression; spinal cord lesions are visible in over 80% of people with MS and correlate with clinical disability.^25^ Spinal cord atrophy has become one of the strongest predictors of disability, showing greater association than the number or volume of lesions, especially for progressive phenotypes of MS.^1,26,27^ CSA is also closely linked to measures of upper extremity function.^28,29^ Even though cord atrophy can account for 77% of MS disability progression after 5 years, a surprisingly low number of progressive MS trials have used cord atrophy as an outcome measure.^3^ In 2017, Moccia et al. highlighted the need for technical advancements to better facilitate robust cord atrophy measurements in clinical trials of MS, especially given the strong relationship between cord atrophy and disability.^2^ The ability to derive a clinically relevant measure of cord atrophy from just a standard 3DT1 brain image can provide increased statistical power for progressive MS trials with just brain imaging, which would reduce the required sample size or length of the trial compared to using only standard brain imaging outcome measures. Alternatively, our approach could help reduce the cost of MRI booking time in cases where separate cord imaging is being acquired primarily for the purpose of measuring cord atrophy. In either case, this can make clinically feasible cord atrophy measurements accessible to help improve understanding of progression in MS and faster evaluate the efficacy of potential treatments.

Studies in MS brains have correlated brain size or “reserve” with resilience to MS pathology.^30^ Specifically, larger brain and grey matter volume has been correlated with higher cognitive reserve and better cognitive function in MS.^31^ Characterization of the natural variation in cord CSA can provide an opportunity to investigate the possibility of “spinal cord reserve” and to better distinguish pathological change from natural variation in MS.

Spinal cord images are prone to artefacts caused by cerebrospinal fluid flow, an inhomogeneous magnetic environment, small cord size, and cardiorespiratory motion.^6^ Furthermore, MRI scanner coil changes are often required between collecting both brain and spinal cord images, increasing the scan time and patient discomfort. As a result, brain imaging is usually prioritized over spinal cord, meaning that cord images are often not acquired at all.^1^

In this study, imaging was performed using 8-channel and 6-channel receive coils designed specifically for brain and cervical cord imaging, respectively. Newer equivalent coils often have ∼16–48 channels, leading to improved (reduced) g-factor and therefore higher signal-to-noise ratio, which will allow for improved image quality in the same acquisition time, or equivalent image quality in a reduced acquisition time. Some scanners are also equipped with a posterior spine coil built directly into the subject table, which improves cord-specific imaging, as well as large-FOV brain imaging where the posterior coil can contribute signal. The agreement demonstrated in this study between C1 CSA_brain_ and C2/3 CSA_cord_ should hold in the case of imaging using these more modern coils because the brain and cord imaging will each independently be improved. If anything, we expect the agreement to be improved by the use of brain imaging coils with more channels because they will provide a more comprehensive sensitivity profile across the imaging FOV. This is especially relevant to the edges of the FOV, such as the inferior caudal edge where the cord appears on 3DT1 brain images.

Removing the need for separate spinal cord scanning can drastically reduce the data acquisition time required for cord atrophy measures. This time-savings is compounded for most studies, where spinal cord imaging increases total exam time due to the data acquisition itself as well as the time necessary to change between head- and cord-specific imaging coils. One exception is for imaging with a combined head and neck coil, designed to perform both brain and cervical cord imaging using a single coil array, without necessitating a coil change. However, for these combined coils to fit the head and neck of most of the population, there generally must be fewer coil elements arranged further from the anatomies of interest, which poses a significant disadvantage in terms of g-factor and signal-to-noise ratio compared to an anatomy-specific coil.^22^ For example, a modern 16-channel head and neck coil will only have about half of those channels contributing to brain imaging, a substantial detriment compared to a 32-channel dedicated brain array. So, although a combined head and neck coil can perform brain and cord imaging without a coil change, it provides sub-optimal conditions compared to use of anatomy-specific coils and therefore still motivates the development of a brain imaging surrogate measure for cord atrophy. Reduced scan time has the benefit of improved patient comfort (especially important for imaging patient groups afflicted with physical disability or incontinence) and reduced susceptibility ot motion artefacts (especially important for cord imaging due to the presence of cardiac, respiratory, vascular, and vocal cord motion).^32^

Using C1 CSA from brain imaging as a proxy for C2/3 CSA from cord imaging also opens the possibility of performing retrospective spinal cord atrophy studies on cohorts and previously acquired data that did not include separate spinal cord imaging. Furthermore, our results highlight the value of including 3DT1 brain imaging as standard practice for clinical MRI exams in MS. Through the use of brain imaging derived C1 CSA, more research can now examine the connection between spinal cord atrophy and MS without being limited by availability and feasibility of spinal cord imaging.

## Conclusion

C1 level spinal cord CSA calculated from brain scans is strongly correlated with C2/3 CSA calculated from separate, reference spinal cord imaging. This could prospectively and retrospectively provide studies with a convenient, clinically relevant measure related to spinal cord atrophy, without requiring separate dedicated cord imaging.
